# Immunological Responses to Tetanus and Influenza Vaccination in Donkeys

**DOI:** 10.1111/jvim.70137

**Published:** 2025-05-25

**Authors:** Maciej Perzyna, Jowita Grzędzicka, Dominika Milczek‐Haduch, Izabela Dąbrowska, Michał Trela, Bartosz Pawliński, Olga Witkowska‐Piłaszewicz

**Affiliations:** ^1^ Department of Large Animals Diseases and Clinic, Institute of Veterinary Medicine Warsaw University of Life Sciences Warsaw Poland

**Keywords:** B cells, horses, immunology, monocytes, ROS, T cells

## Abstract

**Background:**

Donkeys are routinely vaccinated with protocols developed for horses, yet species‐specific data on their immune responses are limited.

**Hypothesis/Objectives:**

We hypothesized that donkeys exhibit robust T‐cell‐mediated immunity and regulatory adaptation after vaccination, comparable to horses.

**Animals:**

Thirty‐six healthy, seronegative donkeys (34 mares, 2 stallions), aged 0.5–23 years (median 8 years), from two farms with similar housing and management conditions.

**Methods:**

Prospective study. Animals were selected based on clinical health assessment and confirmed seronegativity for tetanus and equine influenza. All received a multivalent vaccine containing tetanus toxoid and equine influenza antigens. Blood samples were collected at baseline, 1 month, and 2 months after vaccination. Flow cytometry assessed CD4+, CD8+, and CD4 + FoxP3+ T cells (primary outcomes), and monocyte subsets and B lymphocytes (PanB/CD21+) with intracellular IL‐10, IL‐17, and Ki67 (secondary outcomes). ANOVA with Bonferroni correction (*p* < 0.05) was used for statistical analysis.

**Results:**

CD4+ T cells increased from 25.1% ± 1.4% to 37.3% ± 0.7% at month 1, CD8+ from 20.6% ± 1.5% to 32.2% ± 0.9% at month 2 (*p* < 0.001). CD4 + FoxP3+ peaked at 11.7% ± 0.6% at month 1 (baseline 6.8% ± 0.8%), then returned to baseline. CD14 + MHCII+ and CD14 + MHCII− monocytes declined; CD14 − MHCII+ increased (*p* < 0.01). PanB/CD21+ cells decreased from 41.5% ± 1.8% to 29.0% ± 1.0%, with significant reductions in IL‐10+, IL‐17+, and Ki67+ subsets (*p* < 0.001).

**Conclusions and Clinical Importance:**

Donkeys exhibit strong T‐cell and regulatory immune responses after vaccination, supporting the clinical relevance of applying equine vaccination protocols to donkeys.

**Abbreviations**
AHS: African horse sickness
ACTH: adrenocorticotropic hormone
BSA: bovine serum albumin
CD: cluster of differentiation
CR: CellRox
EIA: equine infectious anemia
EIV: equine influenza virus
HIS: heat‐inactivated serum
IM: intramuscular
MHCII: major histocompatibility complex class II
PBMCs: peripheral blood mononuclear cells
PBS: phosphate buffered saline
PMA: phorbol 12‐myristate 13‐acetate
POMC: proopiomelanocortin
ROS: reactive oxygen species
TRH: thyrotropin‐releasing Hormone
Tregs: regulatory T cells

## 1 Introduction

The primary objectives of veterinary vaccines are to enhance the health and well‐being of companion animals, cost‐effectively boost livestock production, and prevent the transmission of diseases from animals to humans, whether from domestic animals or wildlife [1, 2]. Vaccination is key in controlling infectious diseases across species, including equids. While horses' immune responses to vaccines have been extensively studied [3, 4, 5, 6, 7], limited research exists for donkeys ( *Equus asinus* ), particularly at the cellular level.

Donkeys are often neglected in preventive care, including vaccination [2]. Vaccination protocols are typically adapted from horses, yet donkeys' unique physiological and immunological traits result in different vaccine responses, as seen with hepaciviral infections, Equine Infectious Anemia (EIA), and Equine Influenza Virus (EIV; [8, 9]). Suboptimal immunity occurs when vaccination protocols fail to align with donkeys' specific needs [3, 6, 10, 11, 12, 13], increasing their vulnerability to disease. It is important because suboptimal immunity occurs when vaccination practices, such as incorrect timing or dosage, do not fully align with manufacturer guidelines or immunological needs [10]. This results in insufficient immune protection, leaving animals vulnerable to disease outbreaks.

The immune response to vaccination involves the activation of both innate and adaptive immune cells, such as dendritic cells, monocytes, macrophages, T cells, and B cells, which generate antigen‐specific antibodies [14, 15]. Vaccines stimulate the production of specific antibodies and memory cells, which help protect the human or animal from future infections. Tetanus, caused by *Clostridium tetani* toxin, and equine influenza, a highly contagious viral disease, pose relevant health risks [11, 16]. Vaccination is essential as it prevents these potentially fatal diseases by priming the horse's immune system, ensuring faster and more efficient immune responses upon exposure. However, most studies performed on horses are mostly focused on immunoglobulin production measurements [17, 18], acute phase proteins reaction [19], or clinical evaluation [17]. There are only several studies examining cellular reactions against tetanus toxoid [20] and recombinant canarypox virus vaccination in horses [21]. In donkeys, studies examining cellular responses do not exist, leaving critical gaps in understanding their immune cell responses to vaccines.

This study aims to address this gap by investigating immune cell responses in donkeys after multivalent tetanus and influenza vaccination. Findings could improve vaccination strategies, optimize immune protection, and mitigate disease risks in this under‐studied species.

## 2 Materials and Methods

### 2.1 Animals

Thirty‐six seronegative donkeys (without prior vaccination), aged between 0.5 and 23 years (median, 8 years), and of both sexes (34 mares, 2 stallions) were selected for this study, comprising 16 miniature and 20 standard donkeys. Ten jennies were in advanced pregnancy (7–11 months), one was in lactation (first week after foaling), and two were 2 weeks after mating. The animals came from two herds from the same region: 16 donkeys from Herd 1 and 20 donkeys from Herd 2. This did not affect the results obtained. All animals were provided with a balanced diet of hay and mineral licks containing micro‐ and macroelements like selenium or zinc. A routine clinical and hematological examination was performed, confirming that the animals were healthy. The clinical examination mirrored the standard assessment performed during every veterinary procedure, and it included heart rate, respiratory rate, mucous membrane color and moisture, capillary refill time, hydration status (evaluated by the time it takes for a pinched skin fold over the point of the shoulder to return to normal), gut sounds, muscle condition, body temperature, and general demeanor. In some cases, limb palpation was also performed to detect any signs of inflammation or discomfort. All animals in herd 1 were vaccinated on the same day, as were those in herd 2. However, the interval between the vaccinations in the two herds was 2 days. To minimize the impact of environmental factors, the animals at day 0, before vaccination, served as their baseline control. This approach reduces variability and allows for a more accurate assessment of the observed changes.

### 2.2 Vaccination Protocol

Commercially available vaccine Equilis Prequenza Te (Intervet International B.V.; LOT A144B01, Netherlands) targeting tetanus and equine influenza (A/equine‐2/South Africa/4/03 50 AU1; A/equine‐2/Newmarket/2/93 50 AU; tetanus toxoid 40 Lf2; as adjuvant—Saponin 375 μg, cholesterol 125 μg, phosphatidylcholine 62.5 μg), was administered intramuscularly (1 mL, IM, in the neck muscles, specifically in the middle third of the neck). The vaccine was administered using an aseptic technique with a needle of 0.8 × 50 mm 21 G. The vaccine was stored in a refrigerator at a temperature of 4°C before usage. The donkeys received an initial dose, after a booster 30 days later, to stimulate a robust immune response as routine protocol used in equine practice.

### 2.3 Sample Collection

The BD Vaccutainer system (BD, Franklin Lakes, NJ, USA) with EDTA tubes for hematological and plain tubes for biochemical examination was used. According to the owner's will to monitor the health of their animals, blood was drawn from the jugular vein at three time points: before vaccination (baseline 0), 30 days after vaccination, and 30 days after the booster. Thus, this research, using the samples collected during the standard veterinary procedure, does not fall under the legislation for the protection of animals used for scientific purposes, national decree‐law (Dz. U. 2015 poz. 266 and 2010‐63‐EU directive). No ethical approval was needed.

### 2.4 Peripheral Blood Mononuclear Cells (PBMCs) Isolation

From the rest of the blood left after the routine hematological evaluation, peripheral blood mononuclear cells (PBMCs) were isolated from EDTA‐treated blood for subsequent immunological testing by density gradient centrifugation (SepMate—Lymphoprep System, Cologne, Germany). After density gradient isolation, PBMCs were washed twice in 2% BSA (Invitrogen, USA) in PBS and frozen in 10% Heat‐Inactivated Horse Serum (HI‐S Thermofisher Scientific, New Zeland) and Dimethylsulfoxide (Thermofisher, USA) and stored at −80°C. After all samples collection, cells were thawed. All cell cultures were performed in RPMI 1640 medium with GlutaMAX containing 10% heat‐inactivated horse serum (Thermofisher Scientific, New Zealand), penicillin (100 IU/mL), streptomycin (100 μg/mL; nonessential amino acids (1%), MEM vitamins (100 μM), sodium pyruvate (1 mM), and amphotericin B (1 μg/mL; all reagents were purchased from Gibco, Life Technologies, Bleiswijk, Netherlands)). Isolated PBMCs (4 × 10^6^) were cultured in the absence or presence of phorbol 12‐myristate 13‐acetate (PMA), ionomycin, brefeldin A, and monensin (eBioscience Cell Stimulation Cocktail (plus protein transport inhibitors; 500X), Invitrogen). All cells were incubated for 6 h at 37°C with 5% CO_2_.

### 2.5 Flow Cytometry

PBMCs were characterized by checking the expression of the surface markers using equine‐specific antibodies or antibodies with documented cross‐reactivity. Markers such as CD4+ (clone CVS4), (Life Technologies, Netherlands), CD8+ (clone CVS21, Life Technologies, Netherlands), FoxP3+ (clone FJK‐16 s, Life Technologies, Netherlands), CD5+ (CVS5, Life Technologies, Netherlands), CD14+ (clone 433 423), MHCII (clone CVS20, Life Technologies, Netherlands), PanB cells/CD21 (clone CVS36, Life Technologies, Netherlands), IL‐10 (clone AF1605, Life Technologies, Netherlands), and IL‐17 (clone 4k5F6, Life Technologies, Netherlands) were measured. The production of reactive oxygen species by isolated PBMCs was measured using the CellRox (CR) Deep Red Assay Kit (Life Technologies, Paisley, Scotland) according to the manufacturer's protocol. The proliferation was measured based on Ki67 expression (Life Technologies, Paisley, Scotland). The protein sequences used in this study were retrieved from the NCBI Reference Sequence database and EMBOSS Needle Pairwise Sequence Alignment (PSA). Sequence homology analysis showed high similarity in extracellular domains between *Equus caballus* and *Equus asinus* for CD4, CD8, CD21/PanB cells, CD14, MHCII, and FoxP3 (Table S1), supporting antibody cross‐reactivity. In‐house validation confirmed distinct, expected population staining patterns similar in horses (Table S2 and Figure S1; [22]).

The flow cytometric analysis was performed using a FACSCanto II flow cytometer and Kaluza 1.5 software (Beckman Coulter, Brea, CA, USA); 10 000 cells of each sample were acquired. Before multicolor staining, the compensation was set using single‐positive cells for each color if necessary.

### 2.6 Statistical Analysis

Statistical analysis was conducted using the OriginPro 2022 software package (OriginLab Corporation, Northampton, MA, USA). To evaluate changes in PBMCs marker expression over time after vaccination, a one‐way repeated measures ANOVA was performed. Pairwise comparisons between the time points were conducted using the Bonferroni test, with significance set at a *p*‐value of 0.05. In cases where the data did not meet the assumption of normality, a non‐parametric Friedman ANOVA test was employed. The Wilcoxon–Nemenyi–McDonald–Thompson test was used for post hoc analysis when the non‐parametric test was applied, with significance evaluated at the 0.05 level.

Outlier detection was carried out using Grubbs' test or linear regression methods before the analysis to ensure data integrity. Visualization of the differences in PBMCs marker expression over time was represented through box plot Figures, which effectively illustrated the median, interquartile range, and any potential outliers for each marker.

Additionally, we used AI—Grammarly to assist with language polishing and ensure clarity and consistency in the text.

## 3 Results

### 3.1 Lymphocytes T

**FIGURE 1:**
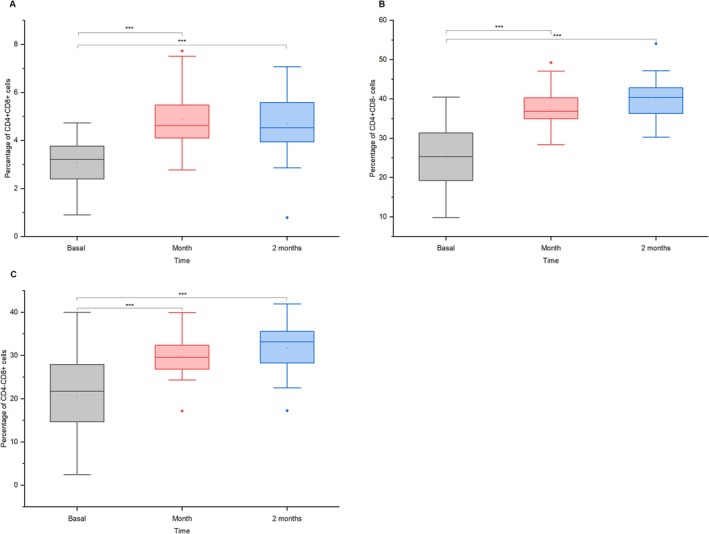
Dynamics of (a) CD4 + CD8+ T cells, (b) CD4 + CD8‐ helper T cells, and (c) CD4‐CD8+ cytotoxic T cells in donkeys at baseline, 1 month, and 2 months after vaccination *** = *p* < 0.001.

The results in Figure 1 demonstrate significant immunological responses in donkeys after vaccination, with notable changes in T‐cell populations. CD4 + CD8+ T lymphocytes increased from ~3% before vaccination to ~5% after 1 month (mean dif. = −1.83, *p* = 3.48 × 10^−8^, *d* = 0.83, 95% CI = [−2.52, −1.15]), maintaining this upward trend at 2 months (mean dif. = −1.64, *p* = 5.70 × 10^−7^, *d* = 0.74, 95% CI = [−2.32, −0.95]). Similarly, CD4 + CD8‐ helper T cells rose from 25% to 37% after 1 month (mean diff. = −12.21, *p* = 8.66 × 10^−12^, *d* = 1.04, 95% CI = [−15.72, −8.70]) and 39.5% after 2 months (mean diff. = −14.37, *p* = 1.85 × 10^−14^, *d* = 1.23, 95% CI = [−17.88, −10.86]; Figure 1b), while CD4‐CD8+ cytotoxic T cells increased from 20% to 30% in 1 month (mean diff. = −9.20, *p* = 4.12 × 10^−8^, *d* = 0.82, 95% CI = [−12.66, −5.73]) and remained elevated at 32% after 2 months (mean diff. = −11.55, *p* = 5.29 × 10^−11^, *d* = 1.03, 95% CI = [−15.01, −8.08]; Figure 1c).

**FIGURE 2:**
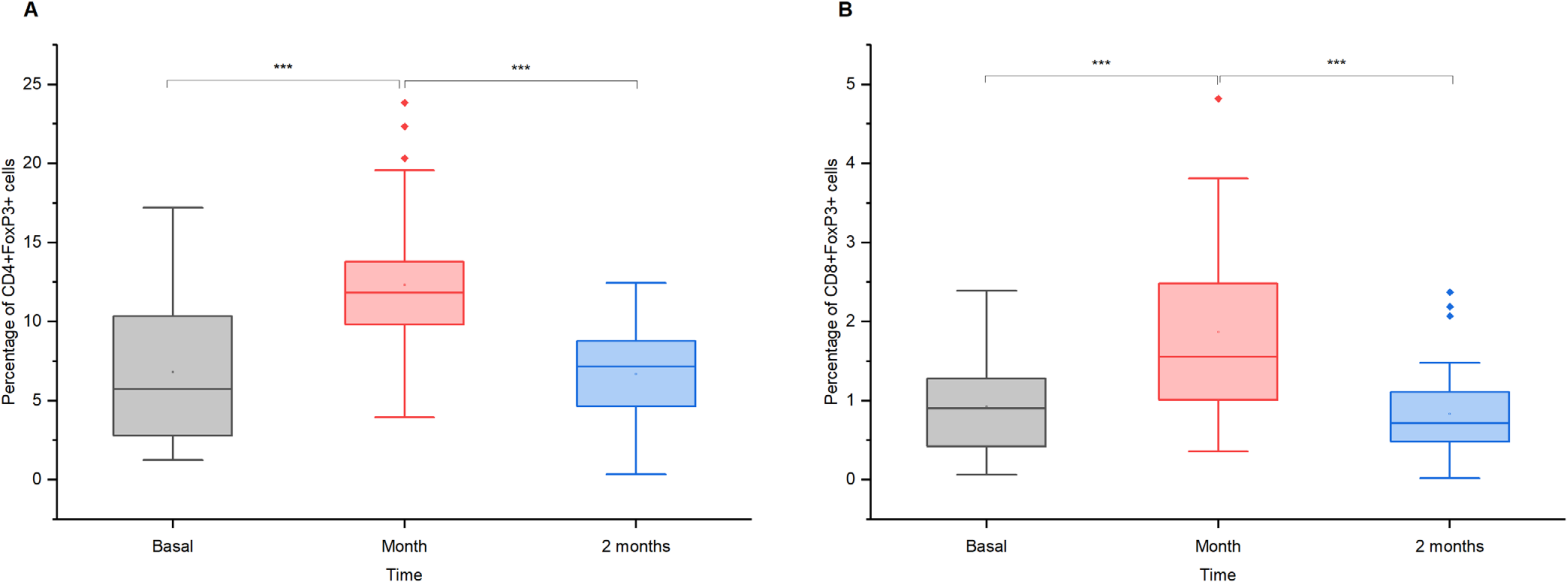
Transient regulatory T cells (a) CD4 + FoxP3+ and (b) CD8 + FoxP3+ responses at baseline, 1 month, and 2 months after vaccination in donkeys *** = *p* < 0.001.

Regulatory T cells (Treg) showed a transient increase after vaccination. CD4 + FoxP3+ T cells rose from 7% to 12% after 1 month (mean diff. = −4.86, *p* = 1.46 × 10^−6^, *d* = 0.71, 95% CI = [−6.99, −2.73]), returning to baseline by 2 months (mean diff. = 0.41, *p* = 1.00, *d* = 0.06, 95% CI = [−1.72, 2.54]; Figure 2a). CD8 + FoxP3+ T cells increased from 1% to 2% after 1 month (mean diff. = −0.95, *p* = 9.48 × 10^−7^, *d* = 0.77, 95% CI = [−1.36, −0.55]), then declined to before vaccination levels (~0.7%) by 2 months (mean diff. = 0.23, *p* = 0.493, *d* = 0.19, 95% CI = [−0.17, 0.64]; Figure 2b).

**FIGURE 3:**
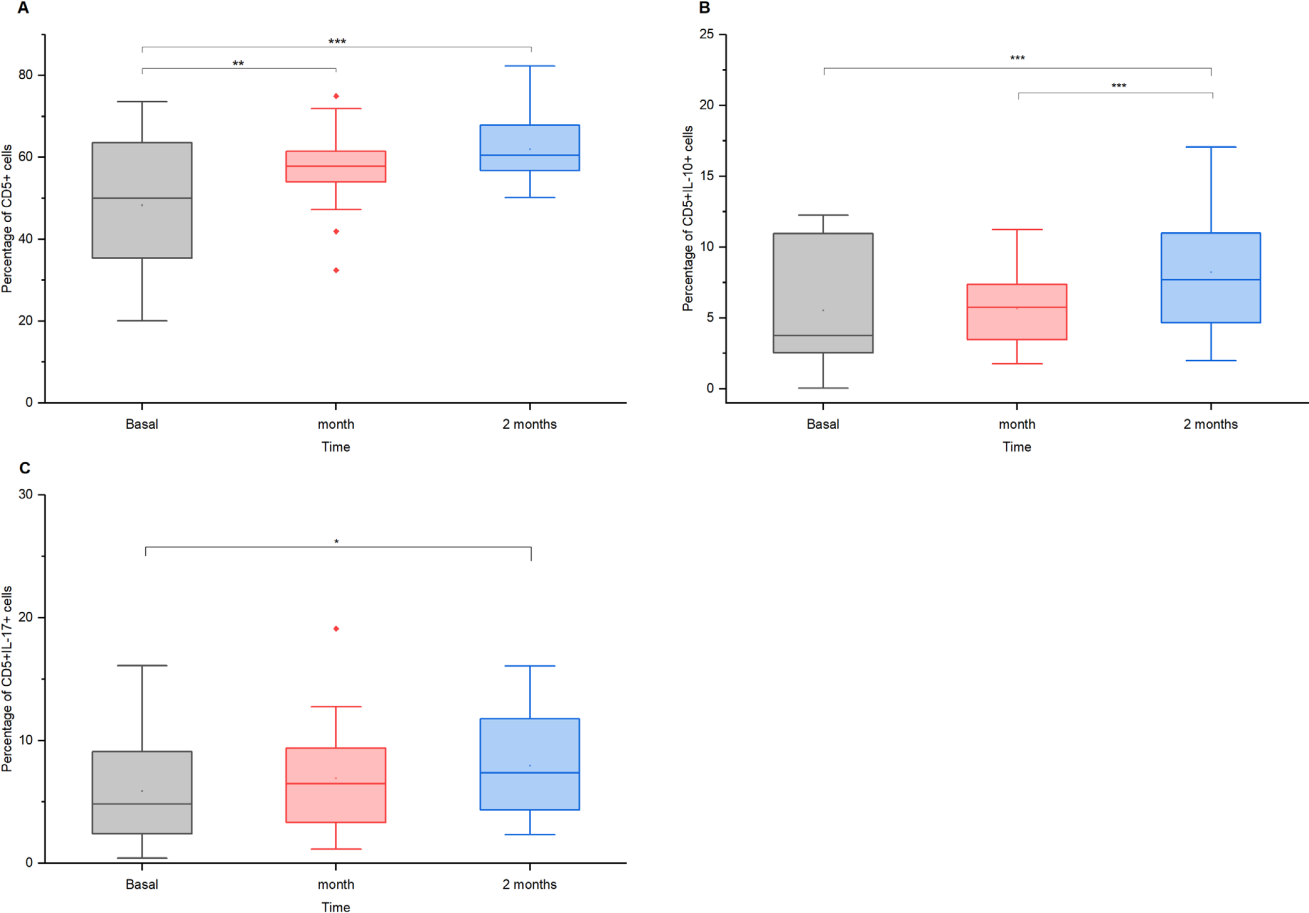
CD5+ T cell activation and IL‐10 and IL‐17 cytokines production at baseline, 1 month, and 2 months after vaccination in donkeys * = *p* < 0.05, ** = *p* < 0.01, *** = *p* < 0.001.

Figure 3a shows a consistent rise in CD5+ T‐cell populations in both one (mean diff. = −9.73, *p* = 0.0030, *d* = 0.43, 95% CI = [−16.66, −2.79]) and 2 months (mean diff. = −14.24, *p* = 1.17 × 10^−5^, *d* = 0.63, 95% CI = [−21.17, −7.31]). IL‐10‐producing CD5+ T cells increased significantly at 2 months (mean diff. = −2.80, *p* = 9.77 × 10^−4^, *d* = 0.48, 95% CI = [−4.60, −0.99]; Figure 3b), while no effect has been observed after 1 month (mean diff. = 0.04, *p* = 1.00, *d* = 0.01, 95% CI = [−1.77, 1.85]). Pro‐inflammatory CD5+ IL‐17+ T cells showed a slight rise after 1 month (mean diff. = −0.85, *p* = 0.584, *d* = 0.12, 95% CI = [−2.90, 1.20]), and a further and statistically significant rise after 2 months(mean diff. = −2.09, *p* = 0.045, *d* = 0.30, 95% CI = [−4.14, −0.04]; Figure 3c).

**FIGURE 4:**
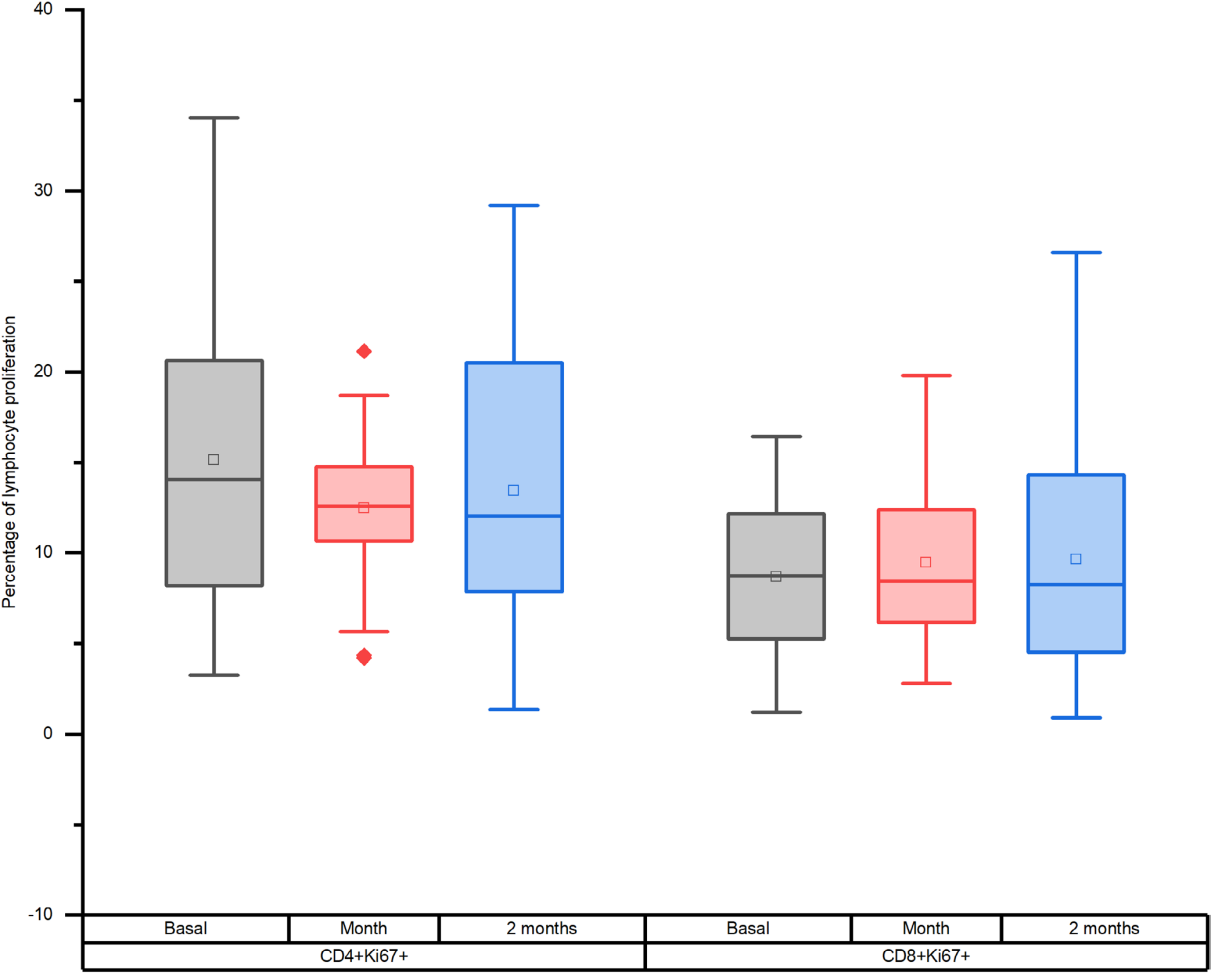
Proliferative response of (a) CD4 + Ki67+ and (b) CD8 + Ki67+ T cells after vaccination baseline, 1 month, and 2 months after vaccination in donkeys.

Lastly, Figure 4 indicates no significant changes in the proliferation of CD4 + Ki67+ (1 month: mean diff. = 2.89, *p* = 0.367, *d* = 0.19, 95% CI = [−1.65, 7.42]; 2 months: mean diff. = 2.22, *p* = 0.702, *d* = 0.15, 95% CI = [−2.32, 6.75]) and CD8 + Ki67+ lymphocytes in response to immunization (1 month: mean diff. = −0.50, *p* = 1.000, *d* = 0.05, 95% CI = [−3.96, 2.95]; 2 months: mean diff. = −0.26, *p* = 1.000, *d* = 0.02, 95% CI = [−3.71, 3.20]).

### 3.2 Lymphocytes B

**FIGURE 5:**
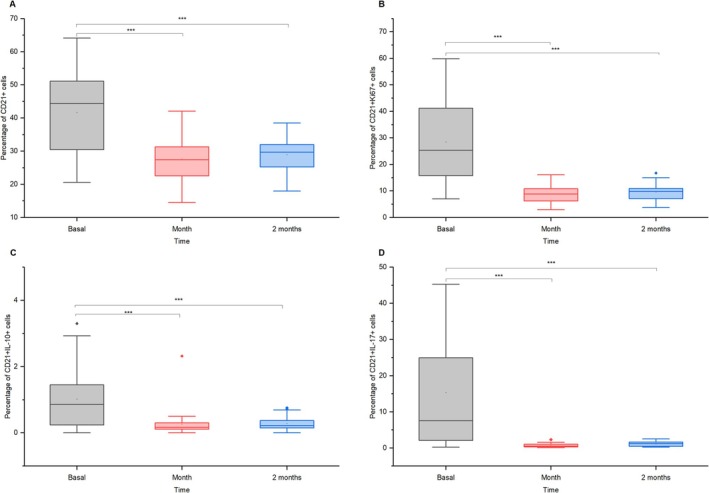
Changes in lymphocytes B (a) PanB cells, (b) Ki67+ proliferating PanB cells, (c) IL‐10‐producing PanB cells, and (d) IL‐17‐producing PanB cells at baseline, 1 month, and 2 months after vaccination in donkeys *** = *p* < 0.001.

After vaccination, lymphocytes B (CD21+) percentages dropped significantly from ~41% to ~29% in 1 month (mean diff. = 14.07, *p* = 3.32 × 10^−10^, *d* = 0.96, 95% CI = [9.57, 18.56]) and ~27% in 2 months (mean diff. = 12.43, *p* = 1.25 × 10^−8^, *d* = 0.84, 95% CI = [7.94, 16.92]; Figure 5a). PanB Ki67+ cells, indicating B‐cell proliferation, decreased from ~28% to ~9.5% in 1 month (mean diff. = 18.93, *p* = 6.39 × 10^−11^, *d* = 1.05, 95% CI = [13.26, 24.61]) and ~ 8.8% in 2 months (mean diff. = 18.23, *p* = 2.13 × 10^−10^, *d* = 1.01, 95% CI = [12.55, 23.90]; Figure 5b). IL‐10‐producing PanB cells declined from 1% to 0.3% in 1 month (mean diff. = 1.08, *p* = 9.48 × 10^−7^, *d* = 0.74, 95% CI = [0.62, 1.55]) and remained low in 2 months (mean diff. = 1.08, *p* = 1.06 × 10^−6^, *d* = 0.73, 95% CI = [0.61, 1.54]; Figure 5c). Similarly, PanB IL‐17+ cells dropped significantly from ~15% to ~1% in 1 month (mean diff. = 14.93, *p* = 2.83 × 10^−9^, *d* = 0.86, 95% CI = [9.77, 20.09]) and ~ 0.7% in 2 months (mean diff. = 14.53, *p* = 6.16 × 10^−9^, *d* = 0.83, 95% CI = [9.37, 19.69]; Figure 5d).

### 3.3 Monocytes

**FIGURE 6:**
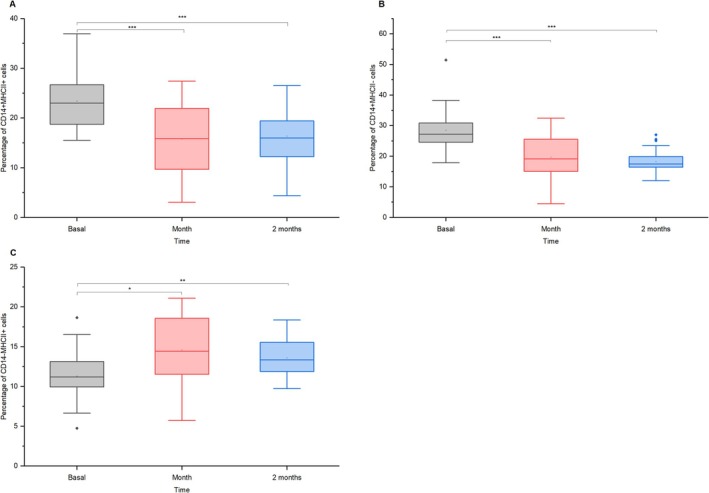
Percentages of (a) CD14 + MHCII+ monocytes, (b) CD14 + MHCII‐ pro‐inflammatory monocytes, and (c) CD14‐MHCII+ monocytes in donkeys at baseline, 1 month, and 2 months after vaccination * = *p* < 0.05, ** = *p* < 0.01, *** = *p* < 0.001.

After vaccination, CD14 + MHCII+ monocytes decreased significantly from ~23% to ~17% in 1 month (mean diff. = 8.71, *p* = 3.81 × 10^−7^, *d* = 0.78, 95% CI = [5.14, 12.28]) and ~ 14.5% in 2 months (mean diff. = 6.38, *p* = 1.38 × 10^−4^, *d* = 0.57, 95% CI = [2.81, 9.95]; Figure 6a), suggesting a lasting change in antigen‐presenting cell activity. Pro‐inflammatory CD14 + MHCII‐ monocytes dropped from ~28.5% to ~19% in 1 month (mean diff. = 9.44, *p* = 1.62 × 10^−7^, *d* = 0.80, 95% CI = [5.70, 13.18]) and ~18% in 2 months (mean diff. = 10.51, *p* = 1.04 × 10^−8^, *d* = 0.89, 95% CI = [6.77, 14.25]; Figure 6b). Conversely, CD14‐MHCII+ monocytes increased from ~11% to ~14% in 1 month (mean diff. = −2.81, *p* = 0.0076, *d* = 0.44, 95% CI = [−4.99, −0.62]) and ~ 13.5% in 2 months (mean diff. = −2.32, *p* = 0.0343, *d* = 0.36, 95% CI = [−4.51, −0.13]; Figure 6c).

**FIGURE 7:**
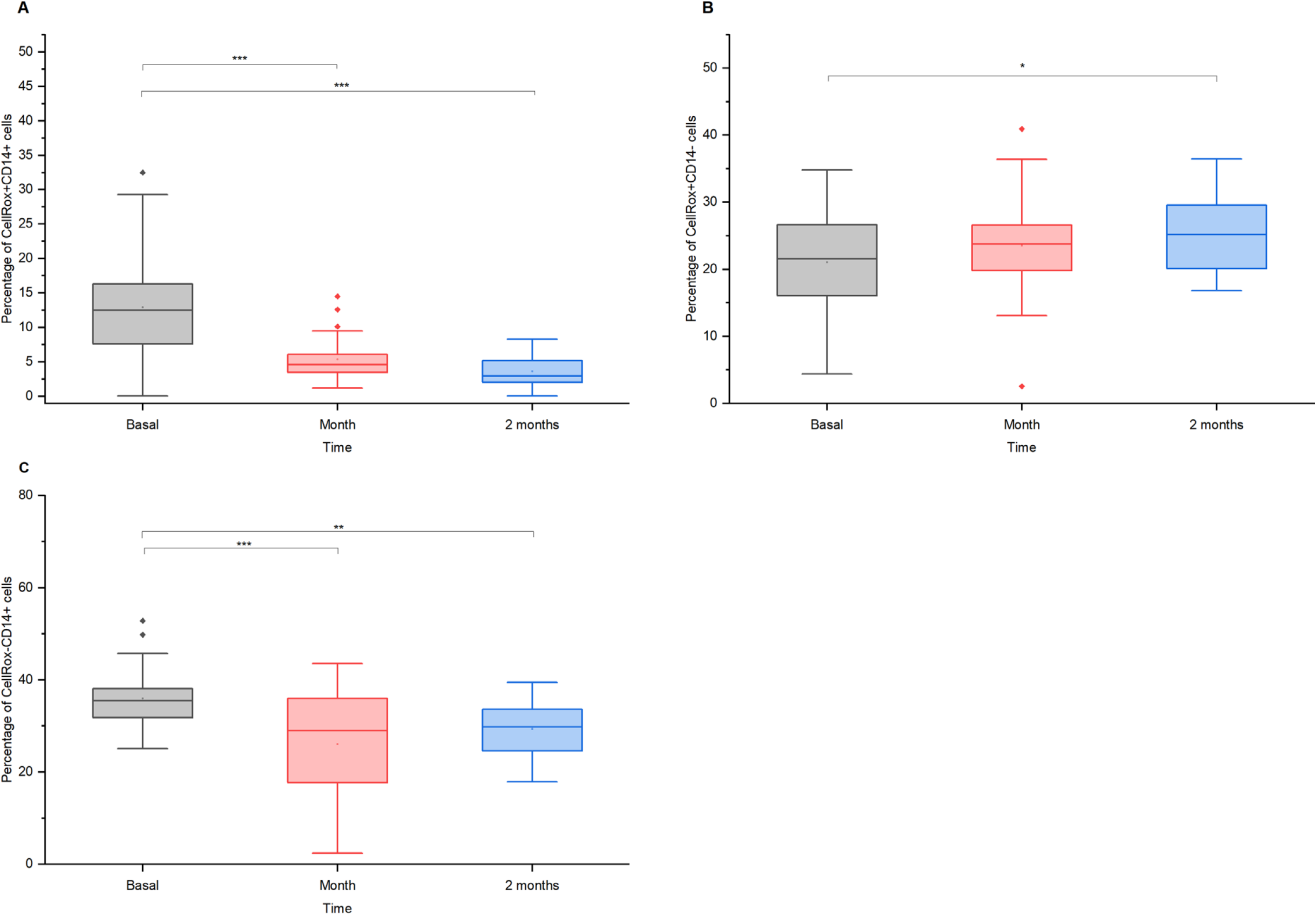
Percentages of (a) ROS‐producing CD14+ monocytes, (b) ROS‐producing CD14‐ monocytes, and (c) non‐ROS‐producing CD14+ monocytes in donkeys at baseline, 1 month, and 2 months after vaccination * = *p* < 0.05, ** = *p* < 0.01, *** = *p* < 0.001.

**FIGURE 8:**
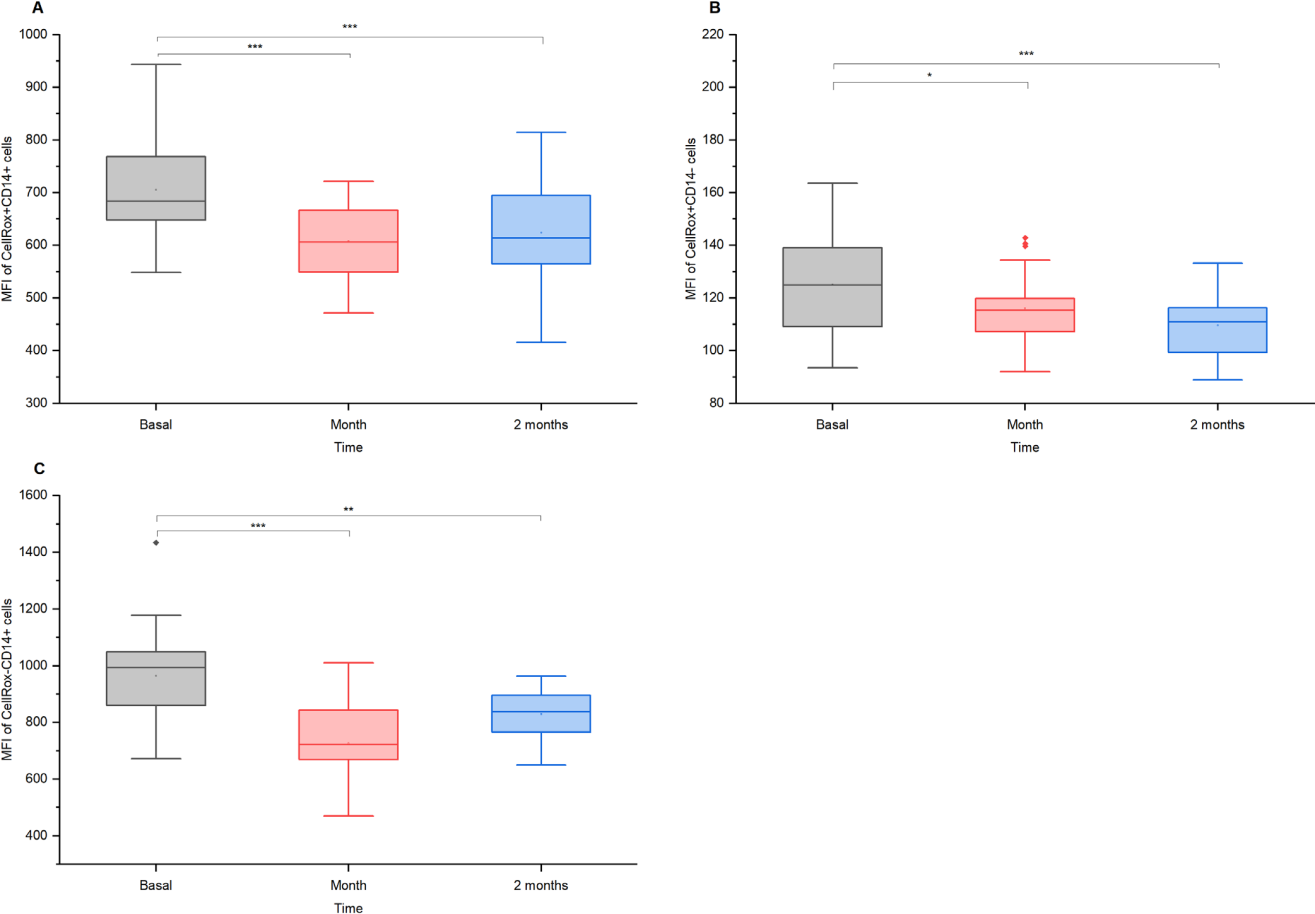
Mean fluorescence intensity (MFI) of ROS production in (a) CellRox+CD14+ monocytes, (b) CellRox+CD14‐ monocytes, and (c) CellRox‐CD14+ monocytes at baseline, 1 month, and 2 months after vaccination in donkeys * = *p* < 0.05, ** = *p* < 0.01, *** = *p* < 0.001.

ROS production in CellRox+CD14+ monocytes decreased from ~13.5% to ~5% in 1 month (mean diff. = 8.13, *p* = 1.59 × 10^−6^, *d* = 0.73, 95% CI = [4.57, 11.68]) and ~3% in 2 months (mean diff. = 10.12, *p* = 7.93 × 10^−9^, *d* = 0.91, 95% CI = [6.57, 13.67]; Figure 7a). While CellRox+CD14‐ monocytes slightly increased after 1 month (mean diff. = −3.74, *p* = 0.085, *d* = 0.29, 95% CI = [−7.85, 0.36]) a statistically significant difference was not observed until 2 months after vaccination, with levels rising from approximately ~21% to ~25% (mean diff. = −4.63, *p* = 0.0219, *d* = 0.36, 95% CI = [−8.73, −0.53]; Figure 7b). CellRox‐CD14+ monocytes dropped from ~36% to ~29% in 1 month (mean diff. = 10.68, *p* = 1.52 × 10^−5^, *d* = 0.63, 95% CI = [5.42, 15.94]), stabilizing at ~25% by 2 months (mean diff. = 6.99, *p* = 0.0053, *d* = 0.41, 95% CI = [1.73, 12.25]; Figure 7c). Mean fluorescence intensity (MFI) of CellRox+CD14+ monocytes declined significantly in 1 month (mean diff. = 102.19, *p* = 3.59 × 10^−4^, *d* = 0.54, 95% CI = [41.12, 163.25]) and remained below baseline in 2 months (mean diff. = 89.99, *p* = 0.0018, *d* = 0.47, 95% CI = [28.92, 151.06]; Figure 8a,b). CellRox+CD14‐ monocytes showed a consistent MFI reduction (after 1 month: mean diff. = 10.15, *p* = 0.036, *d* = 0.33, 95% CI = [0.52, 19.79], and after 2 months: mean diff. = 17.37, *p* = 1.19 × 10^−4^, *d* = 0.57, 95% CI = [7.73, 27.00]). Conversely, CellRox‐CD14+ monocytes decreased in 1 month (mean diff. = 262.07, *p* = 1.35 × 10^−8^, *d* = 0.90, 95% CI = [168.28, 355.86]) and subsequently increased significantly in the second month (mean diff. = −127.57, *p* = 0.0042, *d* = 0.44, 95% CI = [−221.36, −33.78]), though remaining below pre‐vaccination levels (mean diff. = 134.51, *p* = 0.0024, *d* = 0.46, 95% CI = [40.72, 228.30]; Figure 8c).

## 4 Discussion

The present study demonstrated relevant insights into the immune responses of donkeys after vaccination against tetanus and equine influenza. Key findings include robust activation of CD4+ and CD8+ T cells, accompanied by a transient increase in regulatory T cells (CD4 + FoxP3+ and CD8 + FoxP3+) and a notable shift in monocyte subpopulations toward antigen presentation and immune resolution. Furthermore, the observed reduction in B lymphocyte activity after vaccination highlighted a T‐cell‐dominant immune response. These results underscore the immunological mechanisms at play in donkeys and suggest parallels with the immune dynamics seen in horses [20, 21, 23].

In our study, we also used non‐specific cell stimulation as a standard method in immunological research, allowing the evaluation of T cell reactivity independently of the antigen [21, 23]. Also, the effect of tetanus toxoid as a non‐specific stimulator is well‐documented. Thus, we focused on the general cellular response and cytokine production, but further studies, including the assessment of specific antibody levels, are planned to better understand the vaccine's efficacy. However, the evaluation of the cellular immune response provides more detailed insight into early immunological mechanisms after vaccination. Although protective immunity against tetanus and influenza is primarily mediated by vaccine‐induced antibodies, we focused on measuring cellular responses as they arise earlier and reflect the priming capacity of the vaccine, which can be particularly informative in the early after vaccination period [24].

It is important to acknowledge that cellular analysis was conducted on the total lymphocyte and monocyte populations in peripheral blood, which include both naive and primed cells from systemic circulation. Thus, responses cannot be exclusively attributed to vaccine‐specific T or B cells. While environmental antigenic stimulation cannot be ruled out, we controlled variability by maintaining all animals in shared housing and using baseline values as individual controls.

### 4.1 Lymphocyte T

CD4+ T lymphocytes (helper T cells) play a crucial role in the immune response after vaccination. Upon vaccination against diseases like tetanus and influenza, CD4+ cells are activated and begin to proliferate in humans [25] In our study, an increase in the percentage of CD4+, CD8+, CD4 + FoxP3+, and CD8 + FoxP3+ cells 1 month after vaccination suggests a robust immune activation, with CD4+ and CD8+ cells representing helper and cytotoxic T cell responses, respectively, and CD4 + FoxP3+ and CD8 + FoxP3+ cells indicating regulatory T cells (Tregs) that help modulate the immune response. The return of the percent of CD4 + FoxP3+ and CD8 + FoxP3+ cells to baseline values after 2 months suggests that the immune system has successfully controlled the response and returned to a homeostatic state, which is typical in a well‐regulated immune reaction after vaccination, similarly to those reported in humans [26]. This reaction is similar to that reported in horses after tetanus vaccination [20]. CD4+ T cells increased significantly, especially CD4 + CD154+ helper T cells, showing activation after vaccination, peaking around day 14. Thus, the decline in some immune cell populations 2 months after initial vaccination, despite a booster at day 30, likely reflects a typical contraction phase after peak immune activation, with transition toward a regulatory and memory state. This aligns with known after vaccination dynamics in both humans and horses [20].

The percentage of CD4 + CD8+ T lymphocytes increased significantly 1 month after vaccination and maintained the upward trend even after 2 months. CD4 + CD8+ T lymphocytes are “dual‐function” cells influencing both helper (CD4+) and cytotoxic (CD8+) responses [15]. Their increase might indicate the immune system's ongoing adaptation. Worth noting is that they also possess memory phenotypes, aiding in long‐term immune surveillance and rapid responses to antigens.

Additionally, in similar equine studies, CD4+ cells expressed cytokines like IL‐4, TNF‐α, and IFN‐γ, with TNF‐α and IFN‐γ increasing earlier (day 7) and IL‐4 lagging (day 14; 20). In our study, there was an increase in the percentage of CD5 + IL‐10+ cells 2 months after vaccination, suggesting a rise in T cells that produce IL‐10, an anti‐inflammatory cytokine. This indicates the immune system is transitioning toward controlling and limiting excessive immune responses. The subsequent increase in CD5 + IL‐17+ cells implies a shift toward a pro‐inflammatory state, as IL‐17 is involved in recruiting neutrophils and enhancing the immune response to pathogens. This pattern reflects a dynamic modulation of immune responses after vaccination, balancing between activation and regulation. Addams et al. examined the immune response of CD5+ T cells, focusing on their production of IFN‐γ and IL‐2 mRNA expression in response to the EIV in horses [21]. The key results showed that vaccination induced a relevant rise in IFN‐γ‐producing CD5+ T cells and IL‐2 mRNA expression in young horses. In our opinion, examining IL‐17 and IL‐10 might provide a more comprehensive understanding of the immune response in donkeys. IL‐17 is associated with pro‐inflammatory responses, recruiting neutrophils and promoting defense against pathogens [27], while IL‐10 is an anti‐inflammatory cytokine that helps regulate and suppress excessive immune reactions [28]. By studying both, we might be able to evaluate the balance between inflammation and regulation after vaccination. This approach offers insights into how effectively the immune system is controlled after vaccination, preventing both overactivation and under‐response.

Comparing our results connected with donkey immune responses with those of other equids reveals similarities. However, there are only studies comparing T‐cell reactions after vaccination in horses. Thus, a deeper evaluation of horses' and donkeys' immune cell reactions during vaccine stimulation would provide deeper insights into interspecies variations.

### 4.2 Lymphocytes B

There was a decrease in the percentage of B lymphocytes while the percentage of T cells increased after vaccination, possibly because of the immune system's focus on T cell‐mediated responses during this phase. Lymphocytes T aid in the activation of B cells, leading to antibody production, and also help in the activation of CD8+ cytotoxic T cells. Their response is essential for the development of both humoral (antibody‐mediated) and cellular immunity, ensuring a robust and long‐lasting immune defense after vaccination. The initial activation of B cells after vaccination likely occurred earlier, with antibody production and differentiation happening within the first few weeks of the early phase (days to weeks) after vaccination [29]. As the immune response shifts toward regulatory and memory phases, T cells dominate, while B cell activity decreases, leading to a lower percentage of B lymphocytes as the immune response stabilizes [30]. In our study, because blood was obtained during standard veterinary procedures, the earlier time points for blood collection were not performed. However, it adds more detailed information about the immune response in donkeys.

Additionally, we observed a decrease in IL‐10 and IL‐17 production by B lymphocytes, suggesting a downregulation of both regulatory and pro‐inflammatory responses after the initial activation phase after vaccination. A drop in their production could indicate that B cells have shifted away from an active role in modulating these responses as the immune system stabilizes and moves toward a resolution phase after vaccination, focusing more on T‐cell‐mediated pathways. Also, a decrease in Ki67 expression in B lymphocytes was observed, which typically indicates a reduction in cell proliferation. Ki67 is a marker of active cell division, so its decline suggests that B cells are no longer proliferating at the same rate, possibly due to the immune system entering a more regulatory or memory phase after an initial activation [31]. This also indicates that the acute immune response is winding down, and the body is transitioning toward long‐term immunity rather than active B cell expansion.

The activation of T lymphocytes and the deactivation or reduced activity of B lymphocytes one and 2 months after vaccination can be considered a normal immune response pattern [32]. Initially, vaccination stimulates both B and T cells to mount an immune response. As the immune system progresses through its response, T cell activation (such as CD4+ and CD8+ cells) becomes more prominent. B cell activity, which peaks early during antibody production, tends to decrease as the immune system transitions to a regulatory and memory phase, which is part of the resolution of the immune response. This balance between B cell antibody production and T cell memory formation is typical of a well‐regulated immune response to tetanus and EIV vaccination.

### 4.3 Monocytes

Monocytes perform diverse functions, including antigen presentation, phagocytosis, and cytokine secretion. In humans, monocytes are categorized into classical (CD14++CD16−), non‐classical (CD14 + CD16++), and intermediate (CD14 + CD16+) subsets. In horses and dogs, CD14–MHCII+ monocytes resemble human non‐classical monocytes, CD14 + MHCII+ are intermediate, and CD14 + MHCII− are classical, likely applicable to donkeys due to similar immune functions [33, 34].

A decrease in CD14 + MHCII+ and CD14 + MHCII− monocytes, with an increase in CD14‐MHCII+ cells after vaccination, suggests shifts in monocyte differentiation and activation. The reduced CD14+ monocytes might indicate the downregulation of classical subsets, while the rise in CD14‐MHCII+ cells likely reflects increased non‐classical monocytes or antigen‐presenting cells, such as dendritic cells [35]. In horses, dendritic cells were recognized as CD86 + MHCII+ [36]. This aligns with reduced ROS production by CD14+ cells, indicating lower pro‐inflammatory activity. These changes might signify a transition from inflammation and phagocytosis to specialized antigen presentation as the immune response matures. The decline in ROS production and ROS‐positive CD14+ cells could also serve as a protective mechanism to minimize tissue damage caused by oxidative stress [37], supporting a more regulated immune response during the resolution phase.

### 4.4 Clinical Implications

Specific adjuvants that optimize T‐cell activation—such as Alum (aluminum hydroxide), which enhances CD4+ T‐cell activation; MF59 (an oil‐in‐water emulsion used in human influenza vaccines); or CpG oligodeoxynucleotides, which stimulate Toll‐like receptor 9 (TLR9) to boost both T‐ and B‐cell activation [38, 39]—could improve immune responses and promote long‐term immunological memory. Adjustments to booster timing and dosage might also help align more closely with donkey‐specific immune dynamics, thereby enhancing disease protection. While species‐specific vaccine formulations would be ideal, adopting vaccination schedules similar to those used for horses remains a suitable and evidence‐based approach in the current context.

Currently available commercial equine vaccines, which are also used in donkeys, include a wide variety of adjuvants such as aluminum salts, emulsions (e.g., MetaStim, ISA 35), polymers (e.g., carbomers), saponins, and immunostimulating complexes (ISCOMs). Most of these adjuvants are approved for use in horses, and their immunological effects vary in terms of the type and strength of the immune response they elicit. According to the recent review by Carnet et al. [40], aluminum salts are effective in inducing a Th2‐type humoral response but are less efficient in stimulating Th1‐type cellular immunity. Oil‐in‐water emulsions, such as MetaStim, have shown a greater capacity to promote Th1 responses compared to aluminum‐based adjuvants. Carbomer‐based adjuvants, widely used in equine vaccines, appear to activate both humoral and cellular immune responses, although their exact mechanisms of action are still not fully understood. ISCOMs (immunostimulating complexes) and saponins (e.g., QS‐21) have demonstrated the ability to elicit both Th1 and Th2 immune responses, making them promising candidates for the development of future vaccine formulations.

In light of these findings, it appears that current equine vaccine formulations partially align with the recommendations for more immunologically balanced and long‐lasting immune responses. However, data on the performance of these adjuvants specifically in donkeys remain limited. Therefore, while the use of equine vaccines in donkeys is practical and supported by current evidence, their application should ideally be accompanied by further immunological and clinical evaluations to ensure efficacy and safety in this species.

Additionally, our findings suggest that donkeys exhibit a slightly delayed or less robust cellular immune response compared to horses. Therefore, the vaccination protocol used in horses appears suitable for donkeys as well, though administering the booster dose at the lower end of the standard 4–6 week interval might help enhance vaccine effectiveness in this species. In addition, in naïve or high‐risk animals with no prior vaccination history, a third booster dose could be considered to ensure adequate immune protection. At present, we do not recommend altering the antigen dose, as there is insufficient pharmacokinetic or immunogenicity data to support such changes. Based on current clinical experience and our study results, using the same antigen dose as in horses appears both safe and appropriate. Ultimately, optimizing vaccination schedules for donkeys should be based on controlled studies comparing immune response kinetics—particularly cytokine production and antibody titers—under different vaccination protocols, while maintaining principles similar to those applied in horses.

### 4.5 Study Limitations

This study has several limitations. The absence of earlier blood collection points limits insights into the immediate immune response after vaccination. The study focused on immune cell phenotypes but did not include functional assays, such as cytokine quantification or antibody production, which could enhance the understanding of immune function.

Additionally, we acknowledge that the analysis did not target antigen‐specific T or B cells. The immune response observed might partially reflect non‐specific activation or homeostatic shifts. Moreover, minor lymphocyte populations (e.g., NK cells or double‐negative T cells) not included in the antibody panel might explain differences in summed percentages at baseline. Indeed, the ~10% discrepancy between the sum of CD4+, CD8+, and B cell percentages at baseline and at later timepoints likely reflects both the presence of these additional subsets and minor variations in gating stringency during analysis. This technical variation does not affect the main conclusions of the study, which are supported by consistent trends in immune cell responses across all measured timepoints. Furthermore, immune activation after vaccination might enhance antigen expression and improve population resolution in flow cytometry, leading to more accurate identification at later timepoints.

Lastly, while the immune responses observed in donkeys align with those reported in horses, the lack of a direct immunization and assay comparison with horses using the same vaccine limits conclusions about species‐specific differences. Comparisons were instead made with published equine studies to avoid redundant research. Despite these challenges, the research is adequate for exploratory analysis and provides valuable initial insights into donkey immunology.

## 5 Conclusions

This study highlights the unique immune responses of donkeys to vaccination, particularly T cell activation and monocyte phenotype shifts. While an evidence‐based approach is ideal, adopting the same vaccination schedules for donkeys as for horses is acceptable. Our research addresses a critical gap in donkey immunology and can inform more effective, species‐specific vaccination strategies, improving both animal welfare and disease prevention in equids.

## Disclosure

Authors declare no off‐label use of antimicrobials.

## Ethics Statement

This research used the samples collected during standard veterinary procedure. Thus it does not fall under the legislation for the protection of animals used for scientific purposes, national decree‐law (Dz. U. 2015 poz. 266 and 2010‐63‐EU directive). No ethical approval was needed. Authors declare human ethics approval was not needed.

## Conflicts of Interest

The authors declare no conflicts of interest.

## Supporting information


**Data S1.** Supplementary Information.
