# Supplementary material for: Immunological Responses to Tetanus and Influenza Vaccination in Donkeys
Source: J Vet Intern Med. 2025 May 25;39(4):e70137. doi: 10.1111/jvim.70137 (PMC12103835; doi:10.1111/jvim.70137)
Supplement: Supplementary file 1 — Data S1. Supplementary Information. [file JVIM-39-e70137-s001.docx]

**SUPPLEMENTARY**

**Supplementary Figure 1.**
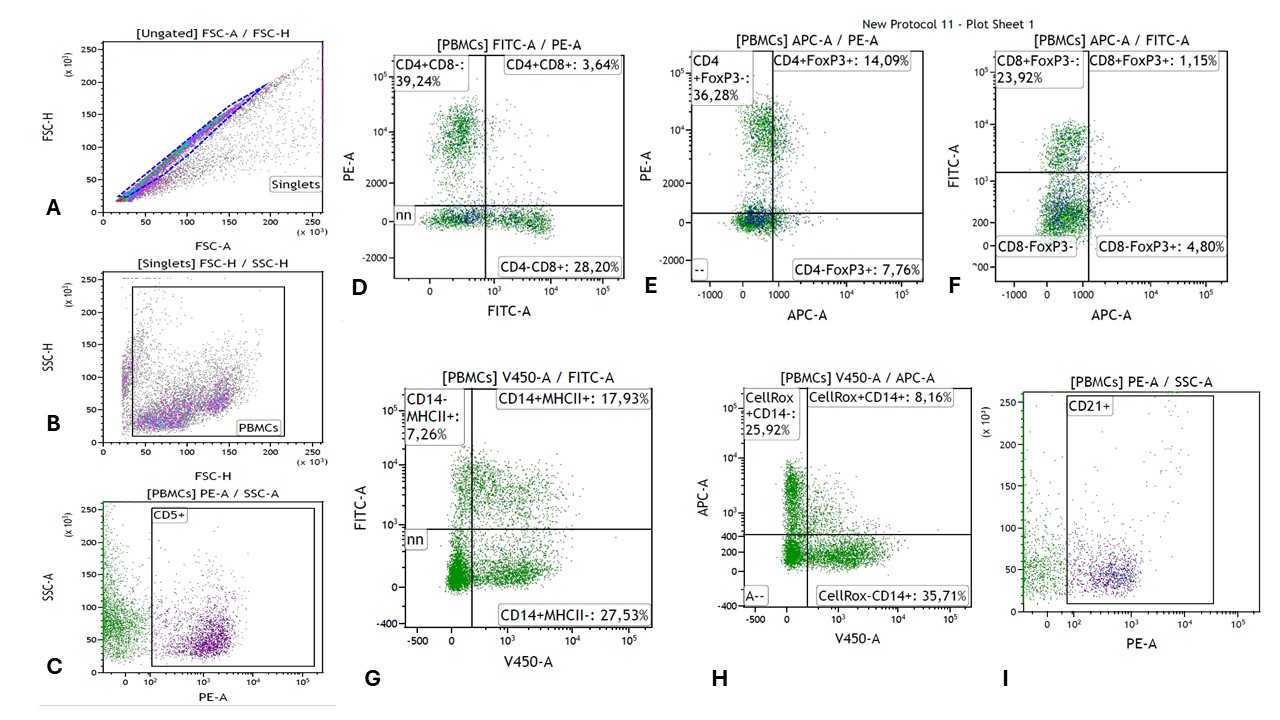
Representative flow cytometry plots showing gating strategy and antibody reactivity in donkey PBMCs; (A) Forward scatter (FSC-A vs FSC-H): exclusion of aggregates (Singlets gate); (B) SSC-H vs FSC-H: PBMC population gating; (C) CD5+ T-cell identification; (D) CD4 and CD8 T-cell subset analysis: CD4+CD8−, CD4−CD8+, CD4+CD8+; (E) CD4+ and FoxP3+ regulatory T cells; (F) CD8+ and FoxP3+ regulatory T cells; (G) CD14+ and MHCII+ monocyte subset distribution; (H) CellRox-based ROS detection in CD14+ and CD14− monocytes; (I) PanBcells – CD21+ lymphocyte identification.

## **Supplementary Table 1.** Sequence homology of selected immune surface markers between *Equus caballus* and *Equus asinus* (EMBOSS Needle Pairwise Sequence Alignment (PSA) &NCBI Reference Sequence database)

| Antigen / Gene | Protein Name | % Identity (Extracellular Domain) | Accession Equus caballus | Accession Equus asinus | Notes |
| --- | --- | --- | --- | --- | --- |
| CD4 | T-cell surface glycoprotein CD4 | 89.0% | XP_070127004.1 | XP_044612091.1 | High identity; major helper T-cell marker |
| CD8A | T-cell surface glycoprotein CD8 alpha | 99.2% | XP_014586534.1 | XP_070367893.1 | Alpha chain of CD8; relevant for cytotoxic T cell identification |
| CD21, Complement receptor type 2 (CR2) | Complement receptor type 2 (CR2) | 95.2% | XP_023496558.2 | XP_014714906.1 | PanB cell |
| CD14 | Monocyte differentiation antigen CD14 | 99.2% | NP_001075396.1 | XP_044636987.2 | Monocyte/macrophage marker |
| MHC class II transactivator isoform X1 | MHC class II, DR alpha chain | 98.8% | XP_023472082.1 | XP_014705404.2 | Antigen presentation molecule |
| FoxP3 | Regulatory T cell transcription factor | 99.8% | NP_001156744.1 | XP_070358268.1 | FoxP3 highly conserved in mammals; equine antibody clone FJK-16s successfully used in multiple species (mouse, horse, human); distinct Treg population seen in donkey samples. |

## **Supplementary Table 2**. Details of antibodies used for flow cytometry in donkey PBMC phenotyping

| Marker | Clone | Supplier | Dilution | Cross-reactivity rationale for Donkey |
| --- | --- | --- | --- | --- |
| CD4 | CVS4 | Life Technologies | 1:10 | Clear CD4+ population; similar to horses |
| CD8 | CVS21 | Life Technologies | 1:20 | CD8+ signal consistent with horse profiles |
| CD5 | CVS5 | Life Technologies | 1:10 | Typical CD5+ T cell staining |
| PanB (B cells) | CVS36 | Life Technologies | 1:10 | Distinct B-cell cluster observed |
| CD14 | 433423 | R&D Systems | 1:10 | Expected monocyte staining |
| MHC II | CVS20 | Life Technologies | 1:20 | Strong APC surface signal |
| FoxP3 | FJK-16s | Life Technologies | 1:10 | Typical Treg staining pattern |
| IL-10 | AF1605 | Life Technologies | 1:10 | Detected IL-10+ subsets |
| IL-17 | 4k5F6 | Life Technologies | 1:10 | Expected IL-17+ profile |
| Ki67 | N/A | Life Technologies | 1:10 | Standard proliferation marker |
| CellRox | N/A | Life Technologies | Per kit | ROS detection according to protocol |
